# The Action of Tobacco

**Published:** 1888-01

**Authors:** 


The Action of Tobacco.—"Observations by Grammatschicow and Ossenkowski have led to the following conclusions: The assimilation of nitrogenous matter is lessened. Digestion is prolonged. The consumption of tobacco hastens the assimilation and elimination of potassic iodide. The influence of tobacco on the processes of nutrition and assimilation is especially noticeable in those just commencing its use.

Walitzkaja has made an examination of more than one thousand workers—men, women, and children—in tobacco, and has, in addition, undertaken a series of experiments on animals, in the laboratory of Prof. Aurep. The workmen, who are constantly exposed to an atmosphere filled with the powder of tobacco, suffered chiefly from nervous symptoms, such as: dilatation of the pupils; cardiac neuroses; exaggerated tendon, and vasomotor reflexes; trembling of the hands; dyspnœa. They are subject to headache, attacks of fainting, gastralgia, and cramps. Next to the nervous system, the respirating organs suffer most frequently. Bronchial and laryngeal catarrh and emphysema of the lungs are common. Phthisis does not appear to be frequent.

Experiments on dogs and rabbits clearly established the fact. The nervous troubles observed in the workmen, are really due to tobacco. The symptoms of poisoning are the same after injection of nicotine as after exposure to an atmosphere impregnated with tobacco; and these symptoms are identical with those observed in workmen."—*Boston Medical and Surgical Journal.*

On the surgical uses of the preparations of lanolin, Güterbrock uses an ointment composed of lanolin and zinc oxide, or iodoform in the proportion of ten to one (10 : 1) in cases of burns, ulcers and artificial eczema. It can be spread uniformly over a greater surface ; its consistency is almost independent of differences of temperature.—*Berliner Klin. Wochenschrift.*

